# A new cryptically dioecious species of bush tomato (*Solanum*) from the Northern Territory, Australia

**DOI:** 10.3897/phytokeys.30.6003

**Published:** 2013-11-27

**Authors:** Christopher T. Martine, David E. Symon, Elizabeth C. Evans

**Affiliations:** 1 Department of Biology, Bucknell University, Lewisburg, PA, USA; 2 1920-2011, Formerly Adelaide Botanic Garden, Adelaide, South Australia

**Keywords:** Bush tomato, Kimberley, new species, Limmen National Park, Litchfield National Park, Northern Territory, *Solanum*, *Solanum cowiei*, *Solanum dioicum*, *Solanum* sp. Litchfield, cryptic dioecy, inaperturate pollen

## Abstract

A new species of dioecious *Solanum* from the Australian “Dioicum Complex” of *Solanum* subgenus *Leptostemonum* is described. *Solanum cowiei* Martine **sp. nov.**, is allied with other members of this problematic lineage, but differs in its slender leaves, limited armature and diminutive habit. The species was first segregated by botanists at the Northern Territory Herbarium as *Solanum* sp. Litchfield (*I.D. Cowie 1428)*; and specimens representing this species have also been referred to by Symon as *Solanum* sp. Fitzmaurice River. Collections suggest that this is an endemic of the sub-arid tropical zone of the Northern Territory. SEM images support initial assumptions that the new species is cryptically dioecious via production of inaperturate pollen grains in morphologically hermaphrodite flowers.

## Introduction

More than three decades ago the late David Symon published the first comprehensive monograph of *Solanum* in Australia (1980), a collection of species descriptions for 125 native and exotic solanums including fourteen species newly described by the author. Symon (1980) included a set of 18 native Australian “spiny solanums” of *Solanum* subgenus *Leptostemonum* within his interpretation of *Solanum* section *Melongena*, thus allying them with the cultivated eggplant (*Solanum melongena*). All nine morphologically androdioecious spiny solanums known in Australia at that time were included in this group. These nine species were later confirmed by Anderson and Symon (1989) to be cryptically dioecious, with morphologically hermaphrodite flowers producing pollen without pores (inaperturate) – thus rendering the flowers (and the plants that bear them) functionally female. The remaining Australian native species included in *Solanum* section *Melongena* sensu Symon are andromonoecious, and were assumed to represent the ancestral condition from which cryptic dioecy arose in the group (Symon 1980, Anderson and Symon 1989) – first by a separation of male and hermaphrodite flowers and then a loss of pollen apertures (Martine and Anderson 2007).

Research using molecular phylogenetics approaches (Martine et al. 2006, Martine et al. 2009) found that the Australian *Solanum* section *Melongena* sensu Symon is not a monophyletic group, but appeared to support the evolutionary pathway from andromonoecy to dioecy in Australian *Solanum*. These studies also identified two clades of dioecious species, 1) the “Kakadu Clade” of The Northern Territory, consisting of *Solanum asymmetriphyllum* and *Solanum sejunctum*, a species described in 2006 (Brennan et al. 2006) and 2) the “Dioicum Complex,” a group of 8-9 described species and several geographic variants/forms with its center of diversity in the Kimberley region of Western Australia, a few species extending into the Northern Territory and one species ranging as far as Queensland.

The Dioicum Complex, in particular, is a taxonomically challenging group, as acknowledged by Symon (1980) and confirmed by phylogenetic studies (Martine et al. 2006, Bohs et al. 2007, Martine et al. 2009). While the clade is well supported, the relationships among the included taxa are difficult to resolve and species boundaries are sometimes blurry – especially in the field. Still, collections and observations made over the last decade by C.T. Martine (CTM) and others (e.g. Barrett 2013) have begun to not only clarify relationships of the Dioicum Complex, but should allow for the formal description of previously unnamed taxa – including the one described herein.

In 2009, CTM followed up on collections by local botanists (including K. Brennan, I. Cowie, D. Lewis, and J. Westaway) of a taxon that had been segregated in the Northern Territory Herbarium as *Solanum* sp. Litchfield (*I.D. Cowie 1428*). New field collections from Litchfield National Park by CTM and colleagues were then used for molecular work (Martine et al. 2011) inferring that *Solanum* sp. Litchfield was closely allied with *Solanum dioicum* and a member of the “Dioicum Complex” sensu Martine et al. (2006).

Unfortunately, few collections had been made of this taxon that included reproductive elements and field surveys made in April-May 2009 and May 2013 located no individuals in flower or fruit. However, multiple new reproductive collections (and some older collections now recognized as this taxon) were deposited/filed at DNA between 2009 and the present day, thus allowing for a species description to now be completed.

Among the specimens now annotated as *Solanum* sp. Litchfield is a sheet that had been in an *indet.* folder until recently. This specimen, *Barritt 1396*, of a staminate plant in flower, was identified by the collector as *Solanum dioicum*. During a visit to the Northern Territory Herbarium in 2004, Symon encountered the specimen, annotated it as *Solanum* sp. Fitzmaurice River, and recorded a page of notes on the morphological characteristics setting this taxon apart from others. Those notes, left in the specimen folder, are here incorporated and used, in part, to describe *Solanum cowiei* – a posthumous contribution described further in Martine (2013).

## Taxonomic treatment

### Solanum cowiei

Martine sp. nov.

urn:lsid:ipni.org:names:77134228-1

http://species-id.net/wiki/Solanum_cowiei

Figs 1 –2

#### Diagnosis.

This species is distinguished from other dioecious Australian solanums by its slender leaves, fine (or absent) armature, and diminutive habit.

**TYPE**: AUSTRALIA. The Northern Territory: Macadam Range, 14°41'07"S, 129°44'39"E, 15 June 2007 (staminate and pistillate flowers; fruit), *Kym G. Brennan 7274* (holotype: DNA! [D0182846]; isotype: PERTH).

#### Description.

**Figure 1.**
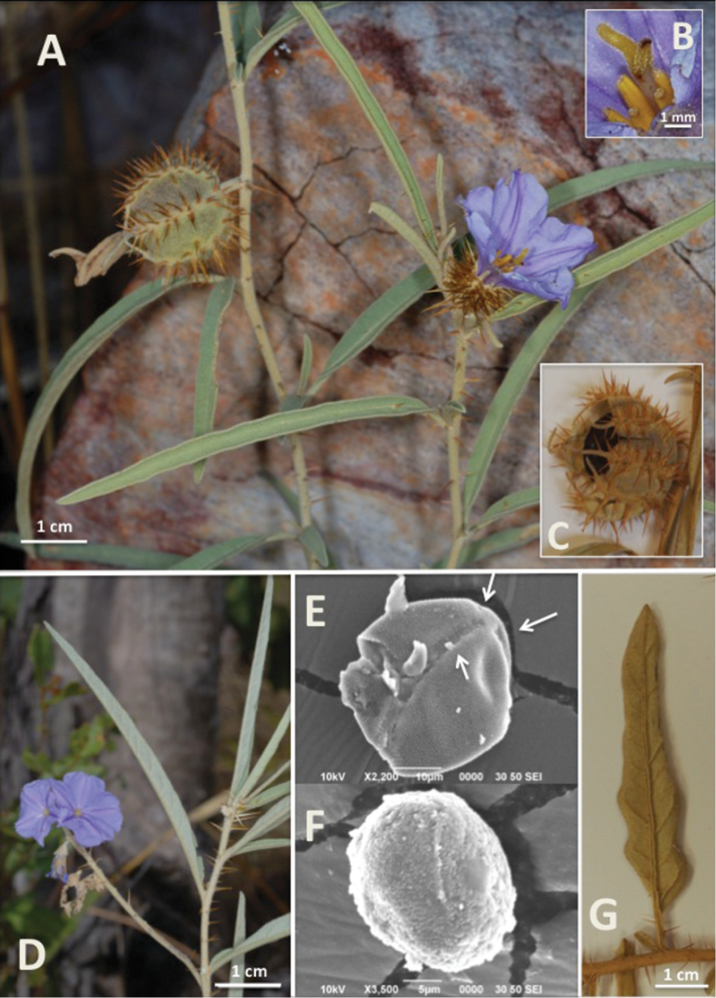
**A** Functionally female plant with morphologically hermaphrodite flower and developing fruit (from type collection, Brennan 7274) **B** Close-up of functionally female flower showing bifid stigma **C** Mature fruit with enlarged fruiting calyx (pressed specimen) **D** Staminate plant in flower (also from type collection) **E** SEM of aperturate pollen grain of staminate flower (from herbarium specimen and partially degraded), arrows showing three germination pores **F** Inaperturate pollen grain of functionally female flower **G** Leaf showing lobing pattern and armed midvein (both infrequent). Photos **A–C** by Kym Brennan. SEM images by Renata Mammone.

Clonal, erect subshrub to 80 cm. Single woody stems 4–5 mm diameter from slender, scarcely-rooted underground stolons, splitting at ca. 40 cm into 2–6 branches. Overall plant aspect yellow-green to gray-green (becoming slightly red-tinged), with older stems eventually woody and gray. Stems with short, dense indumentum of stellate trichomes. Prickles straight, even throughout or slightly widened at base, fine, 5–12mm long, scattered or absent on stems, rarely dense, tending to be absent on woody growth, except near base. Leaves 4–9 cm × 5–10 (-22) mm (largest on newer resprout growth), alternate, linear – lanceolate; margins entire to wavy or rarely lobed; the base tapering to a short petiole 1–1.5 mm long, apex acute; dark green above, slightly lighter beneath, both sides slightly scabrous with short, dense trichomes; trichomes mostly short stalked, porrect-stellate with short central ray. Flowers borne on new growth. *Male* inflorescence a cyme to at least 6 cm long with 9–12 flowers that are shed successively, only 1–2 flowers open at a time; pedicel 5–7 mm, unarmed; calyx 7 mm long with or without a few prickles towards the base, the lobes ending with a slender filiform acumen ca. 3 mm long; petals 5, fused; corolla 1 cm long, purple, broadly stellate to rotate, acumens 0.5 mm; stamens 5, anthers 2–5 mm long, oblong-lanceolate to somewhat tapered, poricidal, filaments 2 mm; in a loose anther cone; ovary, style, and stigma vestigial and not exserted beyond the stamens. Morphologically *hermaphrodite* flowers solitary, functionally *female* with anthers producing inaperturate pollen (Fig. 1F). Female flower on short pedicel; calyx ca. 5 mm, densely armed with long, straight prickles and stellate trichomes; lobes 6 mm, unequal and linear, prickly; corolla ca. 2 cm diameter, 2 cm long, stellate-funnelform, purple; acumens ca. 2 mm; ovary glabrous, 1 mm diameter at anthesis; style erect, 10 mm (including stigmatic surfaces); bifid stigmas 1.5 mm long; stamens of same proportions as in staminate flowers. Peduncle 1.5 cm long, 1.5 mm diameter, sparsely armed with small prickles to 2 mm long. Fruit a green berry 1.2–1.5 cm diameter, globular, drying to black-green and apparently detaching and falling from calyx. Fruiting calyx 2 cm wide, 1.5 cm long, densely armed along sutures, prickles widened (0.5–1 mm) at base and 5–7 mm long, tapering to long fine tip; calyx short stellate-pubescent, more so on sutures and around bases of prickles; calyx lobes extending to slender filiform acumen 5–7 mm long, covered in fine stellate trichomes at tip of each lobe; expanding and surrounding fruit except for ca. 1.5 cm opening at mouth. Calyx retained on stem following fruit drop, at times remaining on plant into next season. Seeds 1.5–2 mm, brown, conspicuously and minutely reticulate.

#### Distribution and ecology.

**Figure 2.**
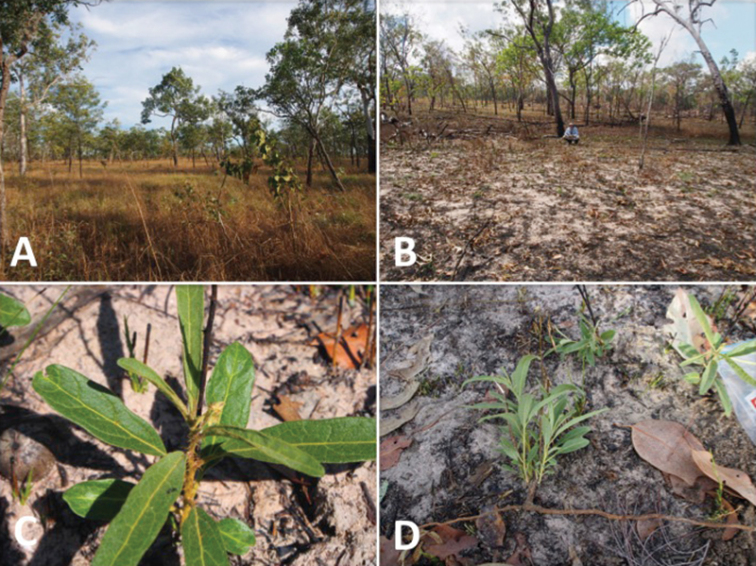
*Solanum cowiei* habitat near the Lost City in **A** unburned condition and **B** burned condition **C** Post-fire resprout growth with deep green color and widened leaves **D** Excavated ramet showing belowground stolon leading to additional ramets of same genet.

*Solanum cowiei* is presently known from a handful of localities in the sub-arid tropical zone of the Northern Territory (a region known colloquially as the “Top End”), most of these habitats are classified under the Tropical eucalypt woodlands/grasslands Major Vegetation Group (National Land and Water Resources 2002). The species is associated with low sandstone outcrops and open eucalypt woodlands, where it typically grows among small boulders or in sandy grassy areas between or around rock formations. The areas where *Solanum cowiei* has been collected are fire-prone and burn at semi-regular intervals (Fig. 2A, B), allowing for this taxon to compete effectively with species of lesser fire tolerance. While the specific pollination biology is unknown, the flowers are clearly buzz pollinated and are likely visited by bees in the genera *Xylocopa* and *Amegilla*, among others (Anderson and Symon 1988). Seed dispersal seems, by initial impressions, to be mechanical. Fruits appear to detach upon maturation, leaving the calyces behind on the plant.

#### Phenology.

Most flowering specimens have been collected from October-November and March-May, with fruiting specimens collected in Jan-Feb and May. Blooming appears to occur on new shoots soon after burns, as evidenced by dozens of male plants found to be in bud within weeks of managed burns set in the Lost City area of Litchfield National Park in late May, 2013.

#### Etymology.

*Solanum cowiei* is named for Dr. Ian Cowie, Chief Botanist at the Northern Territory Herbarium (DNA) and one of the first to recognize the distinct nature of the taxon.

#### Preliminary conservation status.

Based on IUCN Red List Categories (IUCN 2011), *Solanum cowiei* is considered Data Deficient (DD). The species is relatively widespread, occurring over a geographic range of over 800 km, but it has been collected in fewer than 10 localities. The small number of collections, coupled with the fact that populations outside of Litchfield National Park were only confirmed within the last several years, suggest that the species is common in some localities but uncommon on the regional and global scales. The clonal nature of the species is worth considering, as “populations” of *Solanum cowiei* often appear to be large multi-stemmed genets connected by an underground network of stolons. Because the species is dioecious, individual genets cannot self-fertilize, leading to the potential for reproductively isolated clonal individuals. Further data are required before a certain conservation status can be determined. Key populations are protected in Litchfield, Limmen and Keep River National Parks and appear secure given current fire management regimes.

#### Specimens examined.

**AUSTRALIA. Northern Territory:** Bullo River Station, 15°35'22"S, 129°29'11"E, 7 May 2008 (fl), *I.D. Cowie 12068* (DNA); Bullo River Station, 15°40'14"S, 129°37'31"E, 11 May 2008 (fl), *J.O. Westaway 2653* (DNA, NSW); Bullo River Station, 15°39'31"S, 129°34'57"E, 9 May 2008, *I.D. Cowie 12095* (DNA, DREF); Bullo River Station, ca. 25 km NW of homestead, 15°16'15"S, 129°47'23"E, 22 March 2009, *D.L. Lewis 1192* (DNA, AD); Litchfield National Park, Lost City, 13°13.137’S, 130°44.216’E, 26 May 2009 (fr), *C.T. Martine 1753* (DNA, BUPL); Litchfield National Park, Lost City, 13°12'50"S, 130°44'43"E, 8 March 2006 (fr), *J.L. Egan & D. Lucas 5716* (DNA); Litchfield National Park at turnoff to Florence Falls, 13°07’S, 130°48’E, 23 November 1990 (fl), *I.D. Cowie 1428 & C.R. Dunlop* (DNA); Litchfield National Park opposite Florence Falls Rd. turnoff, 13°07'38"S, 130°48'20"E, 20 January 2005 (fl), *J.L. Egan* *s.n.* (DNA, AD); 2 km south of Fitzmaurice River Narrows, 14°49'19"S, 129°58'42"E, 14 May 1994 (fl), I. *D. Cowie 5030 & D.E. Albrecht* (DNA, MEL); Fitzmaurice River, 14°47'28"S, 130°01'04"E, 14 May 1994 (fl), *M.J.A. Barritt 1396* (DNA); Bradshaw Field Training Area, ca. 94 km NW of Timber Creek, 15°02'35"S, 129°52'03"E, 4 April 2007 (st), *B.M. Stuckey & I.D. Cowie 106* (DNA); Keep River National Park, Spirit Hills area, ca. 27 km NW of Bulloo River Homestead, 15°18'38"S, 129°34'12"E, 18 April 2007 (fr), *I.D. Cowie 11692* (DNA, AD); Macadam Range, S of Port Keats, 14°44'00"S, 129°44'00"E, 16 June 2007 (fl), *J.O. Westaway 2368* (DNA); Limmen State Park, St. Vidgeons block, 65 km from ruins, 15°16'46"S, 134°31'03"E, 20 April 2009 (st), *D.L. Lewis 1160* (DNA).

#### Discussion.

*Solanum cowiei* has been known for some time as a local morphotype, having been described by Cowie as *Solanum* sp. Litchfield as early as 2007. In Litchfield National Park, perhaps the most visited recreation area in the Northern Territory, three primary populations are on routes well travelled by day visitors and campers. The most collected population sits along the road to Florence Falls, with tour buses and cars passing on macadam nearly every day within 10 m of individual plants. Thanks to a series of biodiversity surveys undertaken by staff of the NT Herbarium over the last decade or so (e.g., Cowie et al. 2011, Lewis et al. 2010), the species is now known to have a broader distribution spanning from one side of the Top End from Limmen National Park near the Gulf of Carpenteria in the east to Keep River National Park and northward into the Macadam Range in the west. Across this range many characters remain constant, with leaf lobing (unlobed to slightly lobed) and density of prickles (absent to rarely dense) tending to vary the most.

Vigorous post-fire regrowth has been noted in some areas of deep sand beneath open canopies, with one apparently clonal population in a ca. 20 × 20 m area around the Lost City consisting of ca. 40 ramets (Martine, Evans and Dugan pers. obs.). Resprout growth in this grouping was vigorous and well-armed (Fig. 2C), with numerous male flower buds produced on ramets 12–15 cm tall. While other species in this burn area had also begun to resprout, most had not yet developed flower buds – leading one to believe that the flowers of *Solanum cowiei*, once opened, would face little local competition for pollinators. While fire is thought to influence the life histories of other species in the Dioicum Complex (Symon 1980), little data exist on its direct effects on recruitment patterns.

Previous molecular work (Martine et al. 2011) reveals that *Solanum cowiei* is a lineage within the Dioicum Complex – but its relationship to other species in the complex remains unresolved pending continued work in the group (CTM, in progress). Based on morphology, the species appears closely allied to *Solanum dioicum* and *Solanum carduiforme*, the latter species also collected during the Limmen and Keep River surveys. On sight, it differs most from *Solanum carduiforme* in its leaf shape, lobing, and overall growth form. The leaves of *Solanum carduiforme* are wider (5 cm across), “long-triangular” in shape, with well-developed lobes. Compared to all other solanums in the complex, *Solanum cowiei* is rather slight of habit, rarely getting taller than knee height and having poorly developed branching. Aerial shoots, while becoming weakly woody, are much like temporary structures, dying back to the underground stolons during fire or when outcompeted during gaps in the fire cycle – only to spring back to life soon after fires have burned.

In support of its disturbance-adapted nature, much of the biomass in populations of *Solanum cowiei* appears to be below-ground. Stolons function in vegetative reproduction (Fig. 2D), but are also likely important for short-term energy and water storage in the sandy soils inhabited by the species. Stolons unearthed in the field snapped crisply and bore the smell of potato starch. Root systems extending from these stolons are coarse and poorly developed.

The link of this species to fire may be the key determinant of the success of the species in individual sites. In unburned sites surveyed by the first author in the Lost City area of Litchfield National Park (in 2009 and 2013) (Fig. 2A), individual plants were difficult to locate and devoid of reproductive structures. In recently burned areas (Fig. 2B) plants were locally abundant, with budding ramets emerging from the sand at high densities.

The designation of *Solanum cowiei*, along with the recent description of *Solanum zoeae* and allied unnamed variants in the Kimberley region (Barrett 2013), brings the count of cryptically dioecious *Solanum* taxa in Australia to 15. The preponderance of this unusual breeding system in this Australian lineage continues to generate questions regarding the evolution of the condition (e.g., Martine and Anderson 2007) and related ecological interactions (Dugan and Martine 2013, Martine and Evans 2013).

The poor resolution of the relationships among Australia’s dioecious *Solanum* species is a function of both overlapping morphological characteristics (see Symon 1980) and general difficulties in defining lower level relationships across all Old World “spiny solanums” (Bohs et al. 2007, Vorontsova et al. 2013). Further resolution of the relationships among Australian taxa will hopefully be achieved by combining greater sampling of populations (especially in the Kimberley) with Next Generation molecular techniques (C.T.Martine, studies in progress).

## Supplementary Material

XML Treatment for Solanum cowiei
